# Effect of Coenzyme Q_10_ on Insulin Resistance in Korean Patients with Prediabetes: A Pilot Single-Center, Randomized, Double-Blind, Placebo-Controlled Study

**DOI:** 10.1155/2018/1613247

**Published:** 2018-07-29

**Authors:** Ja-Young Yoo, Keun-Sang Yum

**Affiliations:** Department of Family Medicine, College of Medicine, The Catholic University of Korea, Uijeongbu ST. Mary's Hospital, Uijeongbu, Republic of Korea

## Abstract

**Introduction:**

This study aimed to examine whether administration of coenzyme Q10, an antioxidant, improves insulin resistance in patients with prediabetes. The study design was a pilot single-center, randomized, double-blind, placebo-controlled trial.

**Methods:**

This pilot single-center, randomized, double-blind, placebo-controlled trial included a total of 80 adults (aged ≥20 years) with impaired glucose tolerance. After the initial screening visit, subjects were assigned to either the experimental (n = 40) or placebo (n = 40) group via simple randomization. Insulin resistance was represented as the insulin resistance index estimated by homeostasis model assessment (HOMA-IR).

**Results:**

After the 8-week treatment period, the coenzyme group exhibited a significant decrease in the HOMA-IR (P < .001). The free oxygen radical and coenzyme Q10 concentrations were found to correlate significantly (P < .001). However, no significant changes in fasting blood glucose, insulin, and glycated hemoglobin levels were observed in either group. Additionally, no adverse events occurred in either group.

**Conclusion:**

Patients with prediabetes who were administered coenzyme Q10 showed a significant reduction in HOMA-IR values. Therefore, administration of coenzyme Q10 in patients with impaired glucose tolerance may slow the progression from prediabetes to overt diabetes.

## 1. Introduction

The increasing global prevalence of diabetes mellitus has repercussions beyond the individual level [1]; namely, treatment-related complications and diabetes-related mortality incur significant social costs [2]. Therefore, pretreatment efforts to prevent diabetes and reduce the associated morbidity are expected to significantly reduce these social costs. Lifestyle changes (e.g., weight loss) and drug therapies that can reduce the risk of type 2 diabetes mellitus among individuals with prediabetes or those considered at high risk of diabetes are also expected to contribute to a reduction in the risk of cardiovascular disease [3].

The etiology of diabetes is twofold and involves reduced insulin secretion and increased insulin resistance [4]. To date, several clinical methods have been proposed for measuring insulin resistance and beta-cell function, including the homeostasis model assessment (HOMA) model. This popular tool is used to assess beta-cell function (HOMA *β*-cell) or insulin resistance (HOMA-IR) by measuring the plasma fasting glucose and baseline insulin concentrations [5–7].

Hyperglycemia is known to induce oxidative stress via multiple mechanisms. For example, it increases the activity of the polyol pathway, which activates protein kinase C to stimulate the formation of advanced glycation end products [8]. Oxidative stress and the excess production of free oxygen radicals and reactive oxygen species also contribute to the development of diabetes complications and can induce cardiovascular disease [9]. Coenzyme Q10 (ubiquinone), an endogenously synthesized, lipid-soluble antioxidant, is a key component of oxidative phosphorylation in the mitochondria, where energy from carbohydrates and fatty acids is converted to adenosine triphosphate for use in cellular metabolism and synthetic mechanisms. Coenzyme Q10 has also been shown to inhibit cytochrome C [10].

To date, multiple clinical trials have demonstrated the beneficial effects of coenzyme Q10 in patients with cardiovascular disease and hypertension [11, 12], and one study of patients with metabolic syndrome found that an 8-week coenzyme Q10 treatment course had beneficial effects on serum insulin levels and HOMA-IR values [13]. However, the effects of this antioxidant on prediabetic patients with impaired glucose tolerance (IGT) has not yet been evaluated.

Currently, physicians have no active means of preventing the progression of prediabetes to overt diabetes mellitus in a patient and can only advise lifestyle changes [9]. Accordingly, this study aimed to examine the potential effects of coenzyme Q10, an antioxidant, on insulin resistance and insulin secretion consequent to reduced oxidative stress in individuals with IGT.

## 2. Methods

### 2.1. Subjects

**Figure 1:**
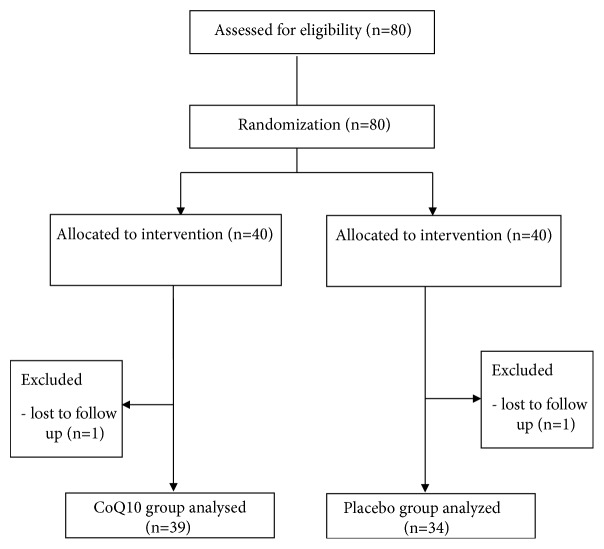
The flow chart of the study.

This randomized, double-blind, placebo-controlled trial included 80 adult patients with IGT aged 20–65 years who attended a university hospital in northern Gyeonggi Province between January 2013 and November 2016. After excluding one subject each from the experimental and control groups who were not measured during outpatient appointments, the data of 78 subjects were included in the final analysis (Figure 1). A diagnosis of IGT was based on the following criterion set by the American Diabetes Association: a 2-hour plasma glucose concentration of 140–199 mg/dL following the oral administration of 75 g of glucose. After the initial screening visit, the participants were randomly assigned to either the experimental or placebo group. The experimental group received coenzyme Q10 (200 mg/day) for 8 weeks, while the placebo group received a placebo via the same schedule. All enrolled participants signed an informed consent form before the initial screening visit.

#### 2.1.1. Exclusion Criteria

Patients were excluded for the following reasons: (i) diagnosis of diabetes or use of oral hypoglycemic agents; (ii) use of vitamins or other antioxidant supplements within the past 6 months; (iii) uncontrolled hypertension (systolic blood pressure ≥160 mmHg or diastolic blood pressure ≥100 mmHg) not treated with antihypertensive medication; (iv) history of cardiovascular disease; and (v) renal insufficiency.

#### 2.1.2. Sample Size

This experimental study aimed to investigate the effects of coenzyme Q10 as the main variable (unit: mg/L). Here, the effect size was expected to be lower than the value of 1.77 suggested by Pavlatoua et al. [14]. The required number of subjects was calculated by simulating an effect size ranging from 0.25 to 1.00 and setting the difference between the control and experimental group to 1, the standard deviation to 1.50, and effect size to 0.67. Using the equation shown below, we would require 36 subjects per group at a significance level of 0.05 and power of 80%. Assuming an expected dropout rate of 10%, we would require 40 subjects per group (80 subjects in total):

(1) $$\begin{array}{c} n=\frac{\left({2\left(z\_\left(\alpha /2\right)+z\_\beta \right)}^{\wedge }2\sigma^{\wedge }2\right)}{{\left(\mu \_c-\mu \_t\right)}^{\wedge }2} \end{array}$$

#### 2.1.3. Double Blinding and Randomization Process

This study was double-blinded; in other words, both the researchers (including the pharmacist, medical staff, and data analysts) and subjects were blinded, and the placebo tablet was identical to the coenzyme Q10 tablet in color, shape, and taste. The participants were randomized using a random number table (i.e., simple randomization).

### 2.2. Ethics Statement

This study was conducted in accordance with the ethical and safety guidelines of the Institutional Review Board (IRB) at the Catholic University of Korea, Uijeongbu ST. Mary's Hospital. This randomized, double-blinded, placebo-controlled trial was registered at the Catholic University of Korea, Clinical Research Coordinating Center (http://eirb.cmcnu.or.kr: UC15HISI0012).

### 2.3. Blood Sampling Procedure and Testing

Venous blood samples of approximately 10 mL in volume were collected before the study and 8 weeks after study commencement. The samples were collected after 12 hours of fasting and used to measure the concentrations of total cholesterol, triglyceride, high-density lipoprotein (HDL) cholesterol, low-density lipoprotein (LDL) cholesterol, fasting blood glucose, fasting insulin, and glycated hemoglobin (HbA1c). The concentrations of total cholesterol, triglyceride, high-density lipoprotein (HDL) cholesterol, low-density lipoprotein (LDL) cholesterol, and fasting blood glucose were measured by using a 7600-IIO (HITACHI, Tokyo, Japan). Fasting insulin concentrations were quantified using the IRMA kit (IZOTOP, Budapest, Hungary), with intra- and interassay coefficients of variance (CVs) of 2.4% and 4.5%, respectively. The glycated hemoglobin (HbA1c) concentration was measured using HLC-723G8 (TOSOH, Tokyo. Japan), with intra- and interassay CVs of <5%.

### 2.4. Measurement of Oxidative Stress

Oxidative stress was measured using blood samples (0.5 mL) collected from the 80 subjects with IGT who provided informed consent for the use of their samples. The free oxygen radicals test (FORT) was used to measure hydrogen peroxide levels at baseline and 8 weeks after study commencement because the amount of free oxygen radicals produced is directly proportional to the amount of lipid peroxides within the sample. The formation of radical molecules via interactions with phenylenediamine derivatives was measured using a spectrophotometer at 505 nm (Form CR 2000, Callegari, Parma, Italy). The results are presented in FORT units, where 1 FORT unit equals 0.26 mg/L of hydrogen peroxide. The FORT intra- and interassay CVs were <5%.

### 2.5. Measurement of Coenzyme Q10

Coenzyme Q10 concentrations (mg/L) were measured at baseline and 8 weeks after study commencement of the study using 0.5-mL aliquots of blood collected from the 80 subjects with IGT who provided informed consent for the use of their samples. All measurements were performed using high-performance liquid chromatography (Agilent 1260, Agilent Tech., Germany), which yielded intra- and interassay CVs of <8.4%. Coenzyme Q10 was separated from lipoproteins via liquid-liquid extraction to methanol and n-hexane, followed by centrifugation and nitrogen gas desiccation. After dissolving the residue in pure ethanol, coenzyme Q10 was detected using a 275-mm ultraviolet light and measured on a C18 reverse-phase analytical column.

### 2.6. Assessment of Insulin Resistance and Secretion

Insulin resistance was assessed using the HOMA-IR. Insulin secretion from pancreatic beta cells was assessed using the HOMA-*β*-cell [7]. Calculations were performed according to the following:

(2) HOMA-IR=Fasting  insulin  μIU/mL×fasting  plasma  glucose  mmol/L22.5HOMA  β-cell=20×fasting  insulin  μIU/mLfasting  plasma  glucose  mmol/L−3.5

### 2.7. Statistical Analysis

Statistical analyses were performed using SAS software, version 9.3 (SAS Institute Inc., NC, Cary, USA). All 79 randomly allocated participants were included in the intention-to-treat (ITT) analysis; one participant with missing data (dropout) was excluded from the ITT analysis. Intergroup differences were analyzed using independent* t*-tests. A multiple regression analysis was performed to determine the effects of coenzyme Q10 administration on parameters of insulin resistance. A P value <.05 was considered statistically significant. We used the independent* t*-test or Wilcoxon's rank-sum test to assess the normality of the variable distributions.

## 3. Results

### 3.1. Characteristics of Subjects

Both groups had male-to-female ratios of approximately 3:1 and mean ages of 49.8 ± 8.4 years in the coenzyme Q10 group and 52.4 ± 6.9 years in the placebo group. No significant intergroup differences in mean height, weight, BMI, or the histories of hyperlipidemia and hypertension were observed. Similarly, the groups did not differ significantly in terms of the baseline total cholesterol, triglycerides, HDL cholesterol, and LDL cholesterol concentrations (Table 1).

### 3.2. Association between Coenzyme Q10 Concentration and HOMA-IR

After the 8-week treatment course, the HOMA-IR value decreased significantly only in the coenzyme Q10 group, and the difference between the two groups was significant. By contrast, however, the HOMA-*β*-cell value did not decrease significantly after 8 weeks in either group. Additionally, the free oxygen radical levels decreased significantly in the coenzyme Q10 group but increased significantly in the placebo group (P < .001) (Table 2). The blood coenzyme Q10 concentration increased significantly in both groups (P < .001). Additionally, the groups did not differ significantly in terms of the fasting blood glucose, insulin, and HbA1c concentrations. As expected, more significant increases in blood coenzyme Q10 concentrations were observed in the coenzyme Q10 group. Additionally, the free oxygen radical concentration increased in the placebo group but decreased in the coenzyme Q10 group, and this difference was significant (P < .001).

A multiple regression analysis of variables potentially affected by changes in the blood Q10 concentration identified free oxygen radicals as a significant factor (P < .001). The first multiple regression analysis confirmed that HbA1c, insulin, and reactive oxygen species, including three variables, were affected by changes in the blood Q10 concentration. A second multiple regression analysis identified four variables, HbA1c, insulin, reactive oxygen, and HOMA-IR, that appeared to be affected by the blood Q10 concentration (Table 3).

Regarding safety, no remarkable complications such as nausea, vomiting, or other gastrointestinal discomfort were observed during the study period.

## 4. Discussion

This study examined whether coenzyme Q10, an antioxidant supplement, could improve insulin resistance in people with prediabetes. Currently, the Korean Food and Drug Administration (KFDA) guidelines recommend a daily intake of 100 mg of coenzyme Q10, with a maximum daily intake of 200 mg [15]. Following a study by Raygan et al. [13], we determined that a daily dose of 200 mg of coenzyme Q10 for a treatment duration of 8 weeks would comply with the KFDA guidelines.

In our study, we observed a significant decrease in the HOMA-IR value over 8 weeks only in the coenzyme Q10 group; additionally, the difference in HOMA-IR values between the two groups was significant. By contrast, no significant reduction in the HOMA-*β*-cell value was observed in either group. Our findings suggest several advantages of coenzyme Q10 administration. Namely, it may defer the progression of glucose intolerance in a patient with prediabetes. Additionally, coenzyme Q10 might represent an innovative strategy for blocking the progression from prediabetes to diabetes, for which no definitive preventive medication currently exists.

Previous work has identified coenzyme Q10 as a regulator of the insulin and adiponectin receptors, tyrosine kinase (TK), phosphatidylinositol kinase (PI3K), and glucose transporters, which suggest that this antioxidant improves insulin sensitivity [16]. In a study of patients with metabolic syndrome, Fariba et al. reported that an 8-week of coenzyme Q10 treatment regimen reduced the insulin concentration and HOMA-IR and HOMA-*β*-cell values. However, the researchers also observed a higher baseline weight in the placebo group, which suggested an inherent difference in insulin sensitivity between the two groups regardless of coenzyme Q10 administration. The possibility that this difference may have contributed to the greater declines in insulin resistance-related values in the coenzyme Q10 group could not be ruled out [13]. Although our study differs from that reported by Fariba et al. in terms of the inclusion of patients with prediabetes and use of high-dose coenzyme Q10 supplementation (200 mg), the finding that coenzyme Q10 administration led to a decline in insulin resistance is comparable.

Although the HOMA-IR is thought to provide a good representation of insulin resistance. The correlation between the HOMA-*β*-cell and actual pancreatic *β*-cell functions remains controversial [7]. In a study of diabetes incidence among in Taiwanese individuals with impaired fasting glucose, the HOMA-IR was found to be an important predictor of diabetes, whereas the HOMA *β*-cell correlated significantly with the incidence of diabetes only after adjusting for the IFG [6]. These results are consistent with our finding that neither subjects in the coenzyme Q10 or placebo group exhibited significant changes in HOMA *β*-cell values, while only the coenzyme Q10 group showed a significant decrease in the HOMA-IR after an 8-week course of coenzyme Q10 supplementation.

In a previous study of insulin resistance and coenzyme Q10 levels, the researchers found that that coenzyme Q10 levels did not significantly vary between obese and normal weight subjects and that coenzyme Q10 did not correlate with insulin resistance (HOMA-IR) [17]. Notably, that study compared the baseline coenzyme Q10 levels and HOMA-IR in a case-control analysis, whereas our study compared insulin resistance before and after coenzyme Q10 administration. Furthermore, Eriksson et al. investigated whether the daily administration of 200 mg of coenzyme Q10 for 6 months would affect glucose metabolism in patients with type 2 diabetes, rather than prediabetes; in contrast to our findings, that study observed no effect of coenzyme Q10 on glucose metabolism [18]. However, coenzyme Q10 was reported to reduce fasting blood glucose levels in another study involving patients with coronary artery disease [19]. These varied findings may be explained by the presence of underlying disease in the coenzyme Q10 group, as well as the coenzyme Q10 treatment dosage and duration.

We note that our study had a number of limitations. First, the subjects' diet histories were not considered because of a lack of data, although we did exclude patients who had taken vitamins or other antioxidants within the previous 6 months. Second, the study duration was short (8 weeks), and specific point of onset of the effects of coenzyme Q10 remains unknown. Although we selected our 8-week treatment period with reference to a study by Raygan et al. [13], a longer period may have generated more significant outcomes. Third, this was a pilot RCT in a single-center setting. RCTs with larger subject populations and longer durations are needed to verify our findings and yield conclusive results. Finally, some of the measurement variables (e.g., triglycerides, insulin) had wide ranges because of the relatively small number of subjects. Despite these limitations, however, this study is meaningful because it established the effects of coenzyme Q10 administration on the HOMA-IR in Korean patients with prediabetes.

In conclusion, coenzyme Q10 administration led to showed significant reductions in the HOMA-IR value, an indicator of insulin resistance in patients with prediabetes. This finding suggests that coenzyme Q10 supplementation may delay the progression from prediabetes to overt diabetes.

## Figures and Tables

**Table 1:** General characteristics of the study subjects.

	Coenzyme Q10 group (n=39)	Placebo group (n=39)	p-value
	mean±SD	mean±SD	
Age (years)	49.79±8.4	52.44±6.9	0.13
Male	29(74.36%)	28(71.79%)	0.80
Female	10(25.64%)	11(28.21%)	
SBP(mmHg)	128.8±10.6	127.1±9.7	0.62
DBP(mmHg)	77.3±11.2	77.3±9.5	0.98
Height(cm)	167.4±8.0	166.9±8.3	0.80
Weight(cm)	75.3±14.9	73.4±14.1	0.60
BMI(kg/m^2^)	26.7±3.7	26.3±3.4	0.74
Hypertension Hx..	20(44.4%)	18(50.0%)	
Dyslipidemia Hx.	25(55.6%)	18(50.0%)	
Total Cholesterol(mg/dL)	183.2±28.5	183.0±28.3	0.97
Triglyceride (mg/dL)	163.5±114.1	130.9±58.6	0.45
HDL-C(mg/dL)	46.1±8.1	50.8±12.2	0.86
LDL-C(mg/dL)	111.5±26.4	112.5±26.3	0.10

SBP, systolic blood pressure; DBP, diastolic blood pressure; BMI, body mass index.

**Table 2:** Changes in biomarkers of insulin resistance and oxidative stress at baseline and 8 weeks.

	Coenzyme Q10 group			Placebo group		
	baseline	8 weeks later	p-value	baseline	8 weeks later	p-value
FBS (mg/dL)	108.5±14.6	106.7±13.5	0.58	110.2±11.24	110.6±14.5	0.87
Insulin (*μ*IU/mL)	20.2±19.9	13.4±10.1	0.19	16.0±20.8	10.5±5.3	0.30
HbA_1c_ (%)	5.9±0.4	6.0±0.5	0.29	6.0±0.5	6.0±0.4	1.00
HOMA-IR	5.5±5.7	3.0±2.5	<.0001	4.5±6.3	3.0±1.6	0.61
HOMA-*β-*cell	153.8±178.6	110.4±101.7	0.29	117.2±128.2	90.6±48.2	0.98
Coenzyme Q10 (mg/L)	0.7±0.3	2.4±1.2	<.0001	0.6±0.2	1.0±0.8	<.0001
Oxygen free radical (Fort unit)	326.5±71.1	247.1±70.4	<.0001	294.8±64.3	323.4±58.0	<.0001

FBS, fasting blood sugar; HbA_1C_, glycated hemoglobin; HOMA-IR, homeostasis model assessment-insulin resistance.

**Table 3:** Univariate and multiple regression analyses of variables that affect changes in the blood concentration of coenzyme Q10.

Variables		Parameter estimate	P-value	Adjusted *r-*square
Univariate regression				
HbA_1c_		-0.0169	.97	-0.0135
Insulin		-0.0039	.56	-0.0089
Oxidative stress		-0.0087	<0.001	0.2860
HOMA-IR		-0.0146	.53	-0.0078
Multitple regression				
	HbA_1c_	0.0023	>.99	
Model 1	Insulin	-0.0027	0.64	0.2397
	Oxidative stress	-0.0089	<0.001	
	HbA_1c_	-0.0331	.93	
Model 2	Insulin	-0.0308	.12	0.2525
	Oxidative stress	-0.0097	<.001	
	HOMA-IR	0.1017	.01	

HbA_1c_, glycated hemoglobin; HOMA-IR, homeostasis model assessment-insulin resistance.

## Data Availability

The data used to support the findings of this study are available from the corresponding author upon request.
